# Oral Teratoma with a Primitive Neuroectodermal Tumor Component: A Case Report

**DOI:** 10.5005/jp-journals-10005-1117

**Published:** 2011-04-15

**Authors:** Swetha Acharya, Amsavardani Tayaar, Sahana Adirajaiah, Gopalkrishnan Kulandswamy

**Affiliations:** 1 Assistant Professor, Department of Oral Pathology, SDM College of Dental Sciences and Hospital, Karnataka, India; 2 Professor, Department of Oral Pathology, SDM College of Dental Sciences and Hospital, Karnataka, India; 3 Assistant Professor, Department of Oral and Maxillofacial Surgery, SDM College of Dental Sciences and Hospital, Karnataka, India; 4 Professor and Head, Department of Oral and Maxillofacial Surgery, SDM College of Dental Sciences and Hospital, Karnataka, India

**Keywords:** Oral teratoma, Small round cell tumor, Primitive neuroectodermal tumor, Childhood.

## Abstract

Teratomas are tumors of germ cell derivation consist of tissues derived from all the three germ cell layers. They comprise the most common extragonadal germ cell tumors (EGCT) in childhood. EGCTs of head and neck region account for only 5% of all benign and malignant germ cell tumors and for 6% of all teratomas. Primitive neuroectodermal tumor (PNET) arising from germ cell tumor is a distinct entity. It develops from the malignant transformation of teratomas along ectodermal lines. This paper presents a rare case of oral teratoma in a 6-year-old male who reported with pain and swelling in the right upper back teeth region of jaw. Under light microscopy, mature and immature structures representatives of trilineage derivatives were appreciated. Sheets of small round cells showing vague rosette and membranous positivity to CD-99 directed us to consider the above diagnosis.

## INTRODUCTION

Teratomas are true neoplasms composed of tissues from all three germinal layers.^1^ It was defined by Willis as ‘true neoplasms composed of multiple tissues foreign to the part in which they arise’.^2^ They are most frequent in the gonads and extragonadal regions like retroperitoneal region, anterior mediastinum, sacrococcygeal, base of the skull, pineal gland, brain and neck.^3^ The most common sites of involvement in head and neck are soft tissues of the neck, thyroid, superficial facial structures, oral cavity, nasopharynx and orbit.^4^ Tera-tomas have a mixture of immature and maturing elements derived from ectoderm, mesoderm and endoderm.^5^ Ectoderm and mesoderm derivatives typically predominate among the immature elements.^6^ They are divided into benign, immature and malignant types depending on the presence and proportion of the immature components.^7^

Teratomas with malignant transformation refer to a form of germ cell tumor in which a somatic teratomatous component becomes morphologically malignant and develops aggressively. They are capable of transforming along all germ layers. The histology of the somatic malignant elements most commonly includes carcinomas and various types of sarcomas; however, there are only a limited number of cases of primitive neuroectodermal tumors (PNET) arising in teratomas reported in the literature. It is supposed that immature neuroepithelium intermingled with other teratomatous elements could be a source of PNET.^8^ We report a rare case of oral teratoma that had malignant transformation of PNET in a young child.

## CASE REPORT

**Fig. 1:**
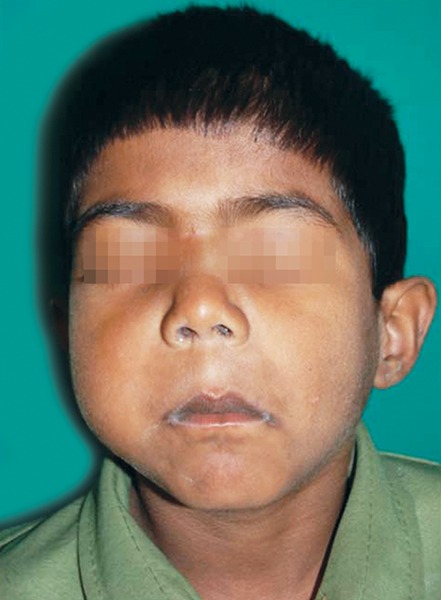
An extraoral view of a solitary, diffuse swelling in the right middle third of face

A 6-year-old boy presented with pain and swelling in right upper back teeth region since a month in 2009. He gave a history of loose tooth in the same region for which the upper right permanent first molar was extracted a month back by a doctor in his place. After the extraction, swelling grew rapidly in size and was also evident extraorally since 20 days. On extraoral examination a solitary, diffuse swelling in the right middle third of face was seen, measuring approx 6x5 cm (Fig. 1). There was also a slight superior displacement of right eye ball. Bilateral submandibular lymph nodes were palpable, soft and tender.

On intraoral examination a solitary, exophytic, irregular growth was seen in the teeth 54, 55 region measuring approximately 4 × 4 cm, obliterating the buccal vestibule and extending palatally. It was fragile, soft in consistency, tender and readily bled on touch.

**Fig. 2:**
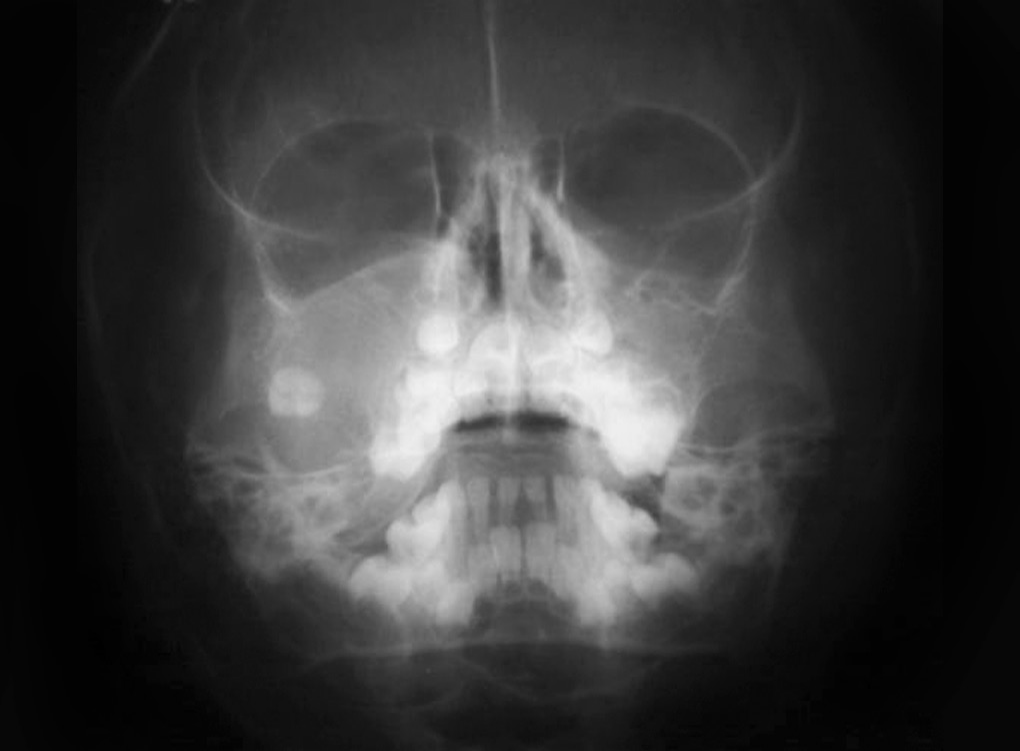
Extraoral radiograph of a lytic lesion in the right maxillary sinus, with the erosion of the lateral wall and displacement of tooth 17 distally

On radiographic examination a lytic lesion was present in the right maxillary sinus, with the erosion of the lateral wall and displacement of tooth 17 distally and an altered level of right infraorbital margin (Fig. 2). Features like rapid growth at the extraction site of 16, palatally displaced premolar clinically, radiographical presence of displaced 17 with erosion of lateral wall and floor of the sinus made us provisionally diagnose the lesion as giant cell lesion or a malignant lesion. Considering the age of the patient and bleeding tendency of the growth, fine needle aspiration cytology (FNAC) was preferred to a biopsy. The smear showed small round cells arranged in small clusters. The cells showed high nuclear to cytoplasmic ratio with scanty cytoplasm, giving an impression of small round cell tumor. Based on FNAC findings, an incisional biopsy was performed under general anesthesia which showed aggregates of blast cells with a thin rim of eosinophilic cytoplasm and a large hematoxyphilic nucleus which was suggestive of small round cell tumor, probably, Ewing’s sarcoma (ES). Following incisional biopsy, the patient’s parents were informed about the aggressiveness of the lesion and advised immediate surgical treatment.

Transiently we lost the patient’s follow up for 4 months, during which patient got the lesion excised twice elsewhere. It was diagnosed as xanthogranuloma/pigmented epulis. After each excision lesion grew rapidly and started bleeding intermittently, indicating an aggressive clinical course. These tissues had been subjected to immunohistochemical (IHC) analysis at a higher center; a diagnosis of embryonal rhabdomysarcoma was given by them.

**Fig. 3:**
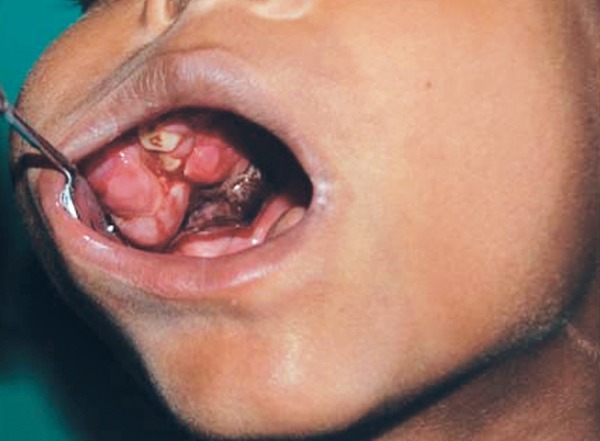
Intraoral view of a pedunculated mass with a lobulated surface

**Fig. 4:**
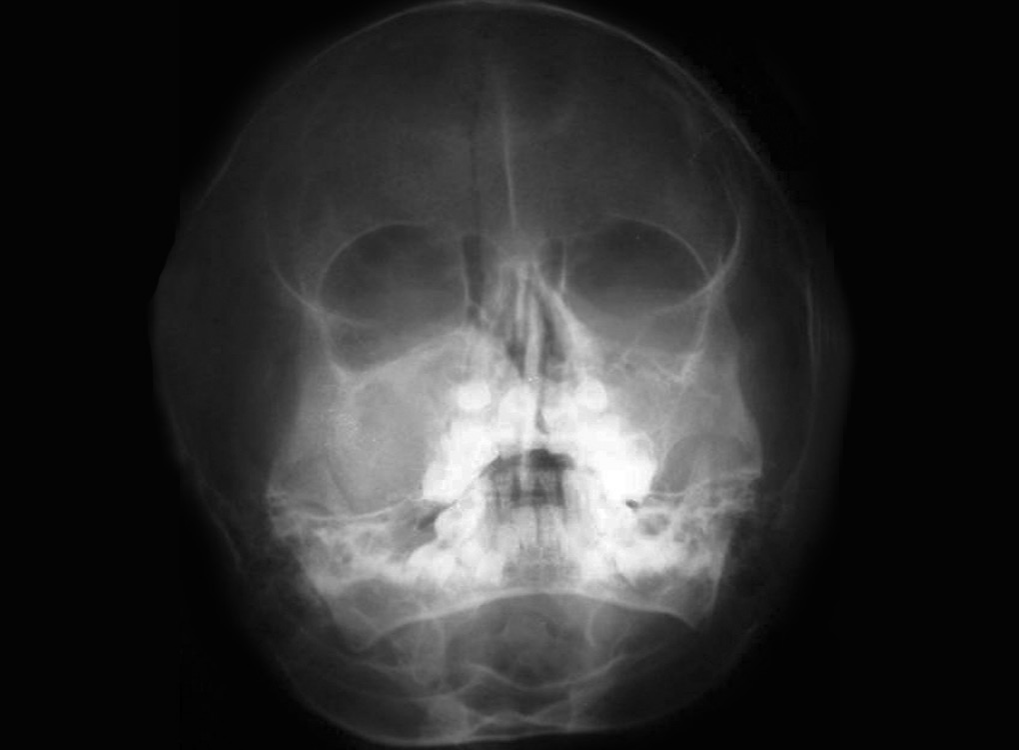
Extraoral radiograph reveals the presence of a lytic lesion of maxillary sinus with thinning out of zygoma

**Fig. 5:**
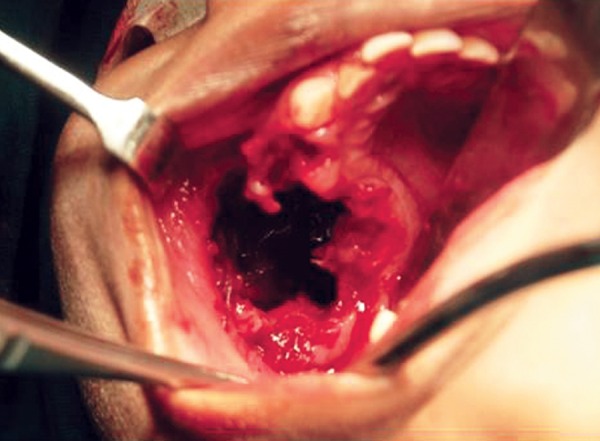
Intraoperative view, after excising the tumor from the walls of the maxillary sinus

The patient presented to our hospital after 4 months with an extensively exaggerated growth in the same site (Fig. 3). A repeat radiographic examination revealed a lytic lesion of maxillary sinus with thinning out of zygoma (Fig. 4). Based on the clinical course and the investigations performed, the patient was treated by excising the tumor from the walls of the maxillary sinus and closing the defect primarily (Fig. 5). Chemotherapy was also advised during the initial phase of recovery. A 1 year follow-up was made which showed no clinical or radiographic evidence of tumor recurrence.

**Fig. 6:**
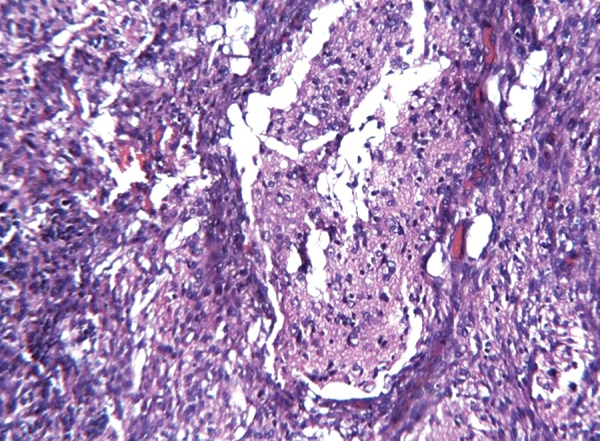
Photomicrograph of glial tissue/neural elements, H&E × 40

**Fig. 7:**
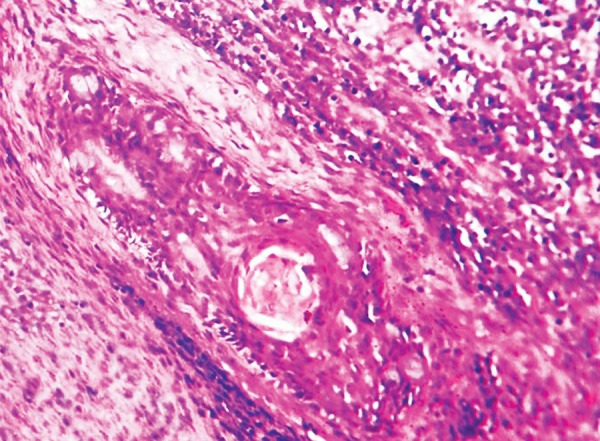
Photomicrograph of squamous epithelial island with mucous metaplasia amidst of small round tumor cells in rows, H&E ×10

**Fig. 8:**
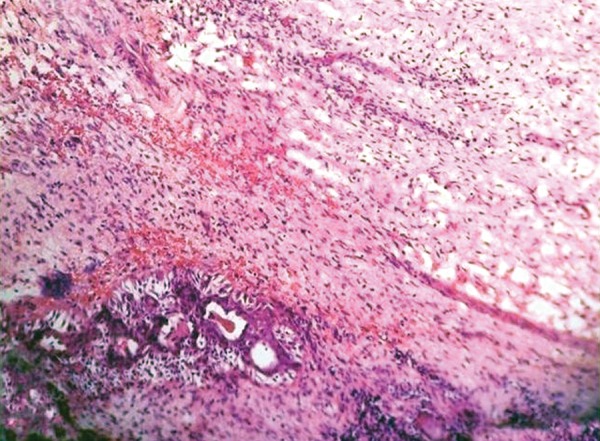
Photomicrograph of an area of ductal differentiation, H&E × 10

**Fig. 9:**
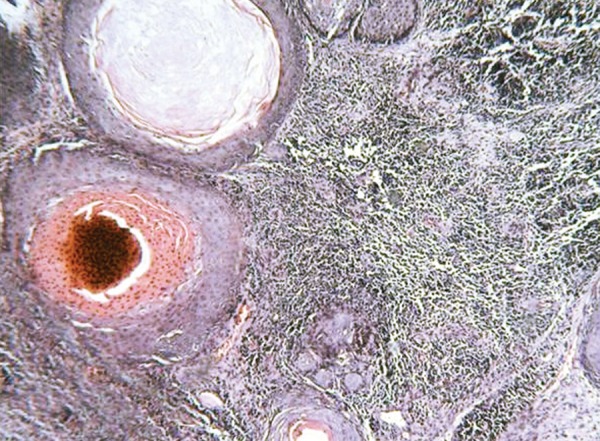
Photomicrograph of areas of squamous differentiation with sheets of small round tumor cells, H&E × 10

**Fig. 10:**
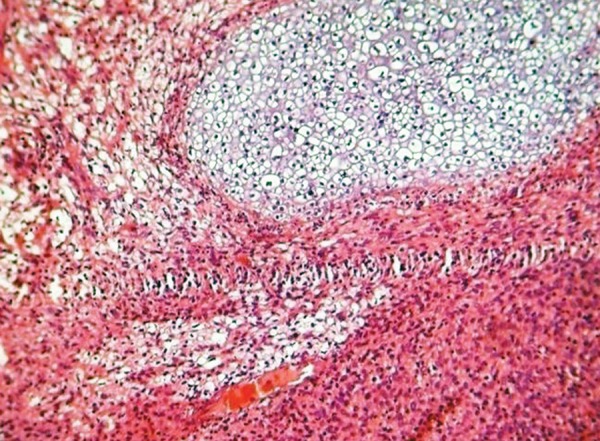
Photomicrograph of cartilage island, H&E x 10

**Fig. 11:**
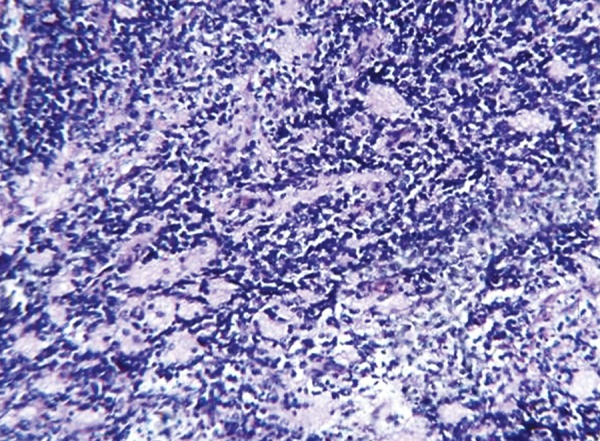
Photomicrograph of small round tumor cells with vague rosettes, H&E x 10

**Fig. 12:**
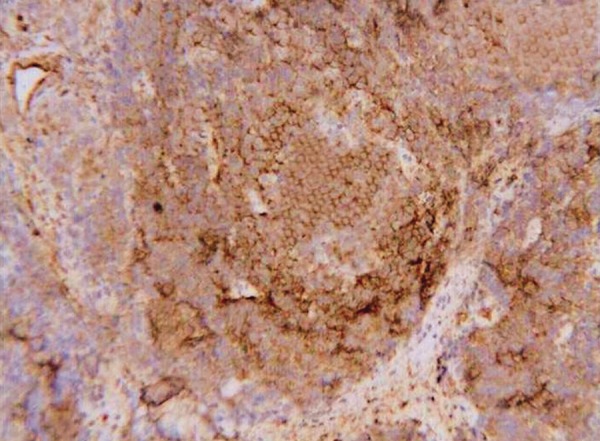
Photomicrograph of membranous CD-99 staining of round tumor cells, x40

The histopathology of excisional biopsy revealed areas showing neural/glial differentiation (Fig. 6), primitive neuroepithelium, myxoid change, isolated areas of mucous metaplasia (Fig. 7), and ductal differentiation (Fig. 8), surplus squamous differentiation (Fig. 9) cartilaginous (Fig. 10) and osteoid differentiation. Apart from the above features, sheets of small round cells tumors showing vague rosettes were also appreciated (Fig. 11). The tumor tissues were subjected to few IHC markers. Sheets of round cells showed membranous CD-99 staining of round tumor cells (Fig. 12), focal positivity of tumors cells for S-100 and epithelial membrane antigen (EMA) positivity was seen in areas of squamous differentiation.

Histological presence of sheets of round cells with vague rosettes and membranous CD-99 positivity depicted primitive neuroectodermal tumor. The presence of surplus squamous, mucous and ductal differentiation characterized ectodermal elements, along with the cartilaginous and osseous differentiation typified the mesodermal/neuroectodermal elements, leading to the final diagnosis of oral teratoma with PNET.

## DISCUSSION

Teratoma (Greek: monstrous tumor) is a tumor of variable maturity and organization. Its elements represent differentiation from all three embryonic germ layers.^5^ In the present case derivatives from various germ layers were observed, like areas of squamous, glandular (ductal and mucous), cartilaginous, osteoid and neural differentiations especially in the excisional biopsy sections.

The presentation of teratomas may be as broad as their histologic appearances.^5^ They are classified as mature, immature and malignant from the standpoint of histologic appearance and future behavior.^3^ They greatly vary in maturity and oncogenic potential.^5^ The synchronous presence of embryonal, fetal and adult elements is possible in a tumor mass and also identifies the level and type of differentiation.^1^ To our knowledge, there are no currently accepted grading criteria for head and neck teratomas. Grading criteria for go-nadal and sacrococcygeal teratomas are well established and hence the same with slight modifications used by Thomson et al, is used for head and neck teratomas.^7^

Immature neuroectodermal elements are the easiest immature tissues to recognize and quantitate.^6^ Teratomas may be classified as mature and immature, based on the presence of immature neuroectodermal elements of histological findings. The survival rate is correlative to the degree of immature components. The risk of recurrence also appears to be related to the degree of immaturity.^9^

Primitive neuroectodermal tumor is one of Ewing’s sarcoma family tumors (ESFTs); defines a group of small round cell neoplasms of neuroectodermal origin that manifest as a continuum of neurogenic differentiation. It is now well accepted that ESFTs constitute a single group of neurally derived neoplasms that share unique immunocytochemical, cytogenetic and molecular markers. The histopathologic features of PNET may resemble the typical undifferentiated ES or atypical ES. However, a neural immunophenotype or evidence of neural differentiation on light microscopic may be helpful in differentiating them. The diagnosis of PNET is mainly based on conventional histologic examination, but sometimes a confident categorization of the tumor may be difficult. CD-99, a glycoprotein product of the MIC2 gene, is present on the cell surface of the vast majority of PNET cases (90%) and therefore represents a useful marker for the diagnosis of PNET, but it is not entirely specific.^9^

Overall, primitive neuroectodermal elements frequently dominate in childhood teratomas and are found more commonly in teratomas of the head and neck region.^10^ Childhood teratomas with neuroectodermal elements may be confused with PNET at histologic examination if there is inadequate tissue sampling.^10^ Teratoma is a pluripotent tissue that can differentiate along ectodermal, endodermal or mesodermal lines with transformation to other cell types such as rhabdomyosarcoma and PNET.^11^ PNET arising from germ cell tumor is a distinct entity. It develops by malignant transformation of teratoma along ectodermal lines. Immature teratomas commonly display foci of immature neuroepithelium intermingled with other teratomatous elements. This immature neuroepithelium could be a source of PNET in many cases.^8^ It is possible that immature teratoma is under recognized in at least some cases because the immature elements are overgrown by PNET, obscuring its true origin.^9^

In general, surgical resection of early lesion is the initial treatment and the best surgical treatment for all these tumors, to prevent recurrences complete surgical excision including a composite adequate margin of uninvolved tissue and subjecting the patient for chemotherapy and radiotherapy along with long-term follow-up.^12^

## CONCLUSION

Clinical presentation with histological form of sheets of small round cells initially led us to think about various small round cell tumors affecting childhood. Sheets of small round cells, presence of vague rosettes and areas of neural differentiation, membranous positivity to CD99, focal positivity to S-100 were in favor of existence of primitive neuroec-todermal tumor. But coexistence of tissues like cartilage, osteoid, myxoid areas, glandular areas (ductal and mucous) and areas of surplus squamous differentiation in various sections directed us to the above diagnosis and considering this diagnosis surgery is not likely to be curative and if advances in survival are to occur, combined modalities of treatment appear to be indicated.
